# Pharmacological effects of *Artocarpus lakoocha* methanol extract on inhibition of squalene synthase and other downstream enzymes of the cholesterol synthesis pathway

**DOI:** 10.1080/13880209.2022.2063346

**Published:** 2022-05-19

**Authors:** Shafaq, Tasleem Akhtar, Hafiz Muhammad Ishaq, Muhammad Shahzad

**Affiliations:** a Department of Pharmacology, University of Health Sciences, Lahore, Pakistan; b Faculty of Veterinary and Animal Sciences, Muhammad Nawaz Shareef University of Agriculture, Multan, Pakistan

**Keywords:** Antioxidant, atherosclerosis, simvastatin

## Abstract

**Context:**

*Artocarpus lakoocha* Roxb. (Moraceae) is reported to possess antioxidant, anti-inflammatory, and anti-skin ageing agents.

**Objective:**

This study evaluates the pharmacological effects of *A. lakoocha* leaves methanol extract on enzymes involved in the cholesterol synthesis pathway in high-fat diet-induced hyperlipidemic rats.

**Materials and methods:**

Twenty-four male Wistar rats, weighing approximately 180–220 g, were divided into four groups: control, diseased (hyperlipidemic), *A. lakoocha* leaves extract treated, and simvastatin treated. The rats were fed with high-fat diet for 2 months to induce hyperlipidaemia, afterward, experimental groups received *A. lakoocha* leaves methanol extract (250 mg/kg) and simvastatin (10 mg/kg) orally until the 89^th^ day of the experiment, while the diseased group continued to receive high-fat diet along with normal saline.

**Results:**

It was found that *A. lakoocha* extract significantly lowered the serum total cholesterol, triglycerides, and low-density lipoprotein (LDL) levels, while effectively increasing serum high-density lipoprotein (HDL) levels as compared to the diseased group (*p* ≤ 0.05). The mRNA expression levels of squalene synthase and HMG-CoA reductase were found to be effectively down-regulated after the treatment with *A. lakoocha* leaves extract (17.45 ± 2.48 vs. 31.91 ± 5.292 and 5.85 ± 3.164 vs. 37.37 ± 6.492) and simvastatin (7.148 ± 0.76 vs. 31.91 ± 5.292, and 3.098 ± 2.09 vs. 37.37 ± 6.492) as compared to the diseased group.

**Discussion and Conclusions:**

The results suggested that *A. lakoocha* leaves extract have observable beneficial effects on inhibition of enzymes involved in cholesterol synthesis pathway and improve lipid profile analogous to simvastatin.

## Introduction

Hyperlipidaemia contributes considerably to the exhibition and development of atherosclerosis and coronary heart diseases (CHDs). Atherosclerosis is a common condition in both developed and developing countries which eventually leads to the development of ischaemic heart diseases (Kumar et al. 2011). Cardiovascular diseases (CVDs) are the leading cause of death globally, an estimated 17.9 million people died from CVDs in 2019, representing 32% of all global deaths (World Health Organization [WHO] 2021).

Primary care physicians face many challenges in the treatment of hyperlipidaemia. Presently, the main treatment for hyperlipidaemia is dietary therapy, quitting smoking, exercise, and medication. Nicotinic acids, statins, and bile acid masking agents are by far the most frequently used medications that can reduce blood lipid and lipoproteins. These medications are useful in preventing many types of CVDs (Zambon et al. 2014). Although they effectively modulate hyperlipidaemia in preclinical and clinical studies, liver and kidney toxicity cannot be ignored (Xu et al. 2014). Many researchers are working to find effective alternative medications for the treatment of hyperlipidaemia. Medicinal plants and their derivatives are gaining popularity due to their versatility, safety, and cost-effectiveness. In recent years, many medicinal plants have received great attention in research for their lipid stabilising effects along with few side effects (Gao et al. 2018).

*Artocarpus lakoocha* Roxb. (Moraceae), commonly called Monkey jack, is broadly dispersed through South and Southeast Asia. All parts of this medicinal plant have diverse medicinal values (Charoenlarp et al. 1981). This plant has many pharmacological activities including anti-inflammatory, antiviral, anticancer, and anti-HIV (Pandey and Bhatnagar 2009; Peng et al. 2018; Islam et al. 2019). ‘Ma-Haad’ is the common name of *A*. *lakoocha* in the Thailand region; the heartwood is used to make desiccated aqueous extract known as ‘Puag-Haad’. This extract is customarily utilised as an anti-helminthic drug (Sritularak et al. 2010). Fruit of this plant, containing vitamin C and β carotene is sour-sweet and used as a tonic to the liver. The fruit pericarp of *A. lakoocha* also has antioxidant, antibacterial, insecticidal, and anthelmintic effects (Sein et al. 2009). The seeds are good purgative for children (Maneechai et al. 2009) and relief of stomach and liver diseases (Piyush and Ramesh 2014). Tree bark is used to treat lesions and bark powder for wound healing (Saha 2019). The roots of this plant have shown antibacterial and cytotoxic effects (Likhitwitayawuid et al. 2005). *A. lakoocha* has also been used in Thai remedial agents for anti-inflammatory and anti-skin ageing actions (Povichit et al. 2010). A literature survey revealed that there was no scientific reported work on the antihyperlipidemic activity of *A. lakoocha*.

Therefore, the current study was designed to assess the pharmacological effects of *A. lakoocha* methanol extract on enzymes involved in the cholesterol synthesis pathway in a diet-induced hyperlipidemic rat model.

## Material and methods

### Experimental animals

Twenty-four male Wistar rats (7–8 weeks old, 180–220 g weight) were raised in the animal house of the Department of Pharmacology (University of Health Sciences, Lahore) under standard laboratory conditions and were provided free access to food and water. All experimental protocols have been approved by the Ethical Review Committee, University of Health Sciences, Lahore, application number UHS/REG-20/ERC/3488 and were performed following the Helsinki declaration prepared by the World Medical Association.

### Preparation of methanol extract of *Artocarpus lakoocha* leaves

*A. lakoocha* leaves weighing 2 kg were collected from Lahore district of Pakistan in March 2020 and identified by Dr. Abdul Rehman Khan Niazi from the Institute of Botany, University of the Punjab Lahore, who gave voucher no. 105-1-20. The leaves were dried in shade at room temperature, and soaked in 70% methanol, and intermittently shaken. After filtration, the extract was concentrated under reduced pressure by utilising a rotary evaporator attached with a vacuum pump at 40 °C. The concentrated extract was stored at −20 °C and the dose was prepared by mixing the extract in sterile normal saline (0.9%), just before the administration.

### Experimental design

Rats were allocated into four groups using the random lottery method; i.e., Control, Diseased, AL and Sim (*n* = 6).

### Control group

Rats in the control group were given normal rat chow (Table 1) and water *ad libitum*, starting at day zero of the experiment, and in addition, normal saline was also started orally from the 60^th^ day and continued until the 89^th^ day.

**Table 1.** The formulation of both standard and high-fat diet for rats.

Ingredients	Standard diet (grams)	High-fat diet (grams)
Casein, Lactic, 30 Mesh	200.00	200.00
L-Cystine	3.00	3.00
Starch, Corn	550.00	72.80
Lodex 10	150.00	100.00
Sucrose	4.00	176.80
Solka Floc, FCC200	50.00	50.00
Soybean Oil, USP	25.00	25.00
Fat (Butter oil)	20.00	177.5
Minerals (S10026B)	50.00	50.00
Choline Bitartrate	2.00	2.00
Vitamins (V10001C)	1.00	1.00
Dye	0.05	0.05

### Diseased group

Rats in this group were fed with high-fat diet (Table 1) and water *ad libitum* throughout the experiment. In addition, normal saline was also started orally from the 60^th^ day and continued until the 89^th^ day.

### *Artocarpus lakoocha* leaves methanol extract group

Hyperlipidaemia induced rats were given *A. lakoocha* leaves extract (250 mg/kg) orally, commencing from the 60^th^ day until the 89^th^ day of the experiment.

### Simvastatin group

In this group, hyperlipidaemia induced experimental rats were given simvastatin, 10 mg/kg (Sigma-Aldrich) orally, commencing from the 60^th^ day until the 89^th^ day of the experiment.

#### Induction of hyperlipidaemia

Eighteen animals were fed with high fat diet and water *ad libitum* for 59 days to induce hyperlipidaemia. All ingredients used to prepare the standard and high-fat diet were processed according to the (AIN)-93 M method of the American Institute for Nutrition (Alkhudhayri et al. 2021).

#### Treatment protocol

On the 60^th^ day, hyperlipidemic rats were further allocated into three groups having six animals in each, labelled as Diseased, AL, and Sim group. These rats were continued on high-fat diet until the 89^th^ day of the experiment. In addition, diseased group was given normal saline orally from the 60^th^ day and continued till the 89^th^ day. The AL group was given 250 mg/kg body weight dose of *A. lakoocha* leaves extract orally and the Sim group was given simvastatin at the dose of 10 mg/kg body weight orally (Sikarwar and Patik 2011) until 89^th^ day of the experiment.

### Biochemical analysis

Biochemical parameters including serum total cholesterol, triglycerides, HDL and LDL levels were estimated by using Randox Rx Monza clinical chemistry analyser and commercial kits of Randox UK.

### Estimation of hepatic squalene synthase and HMG-COA reductase expression

Total RNA was extracted by using TRIzol reagent (Merck) following the manufacturer's guidelines and quantified by using NanoDrop ND-1000 spectrophotometer and quality was assessed by calculating the optical density (260 nm/280 nm). Total RNA (1.5 µg) from each sample was reverse transcribed by using Oligo (dT) 18 primer and RevertAid (Thermo Scientific). cDNA made was used as a template for amplification. The primer pairs used were squalene synthase (forward) 5′-TGTTGCTGGACTGGTGGGAA-3′; squalene synthase (reverse) 3′-CTTCACGGCCACATCTACGT-5′, HMG-COA reductase (forward) 5′-AGTGGCAGAAGCCGAGACTT-3′; HMG-COA reductase (reverse) 3′-GTGCGTCTCCATGAGGGTTT-5′ and β-actin (forward) 5′-TGTCACCAACTGGGACGATA-3′; β-actin (reverse) 3′-AACACAGCCTGGATGGCTAC-5′. PCR was performed on Thermo Cycler of Labinet (Model # MultiGene OptiMax) and PCR products were resolved on agarose gel (2%) stained with ethidium bromide and imaged by using BioRad UV ChemiDoc system. The gel was later analysed using Image J software and was normalised to that of housekeeping gene β-actin.

### Statistical analyses

Results were analysed using Graph pad prism 6 by applying one-way ANOVA following Tukey’s *post hoc* test for multiple-comparison. A *p*-value ≤ 0.05 was considered statistically significant. Data was represented in the form of Mean ± SD.

## Results

### Effect of *Artocarpus lakoocha* leaves extract on total cholesterol levels

**Figure 1.**
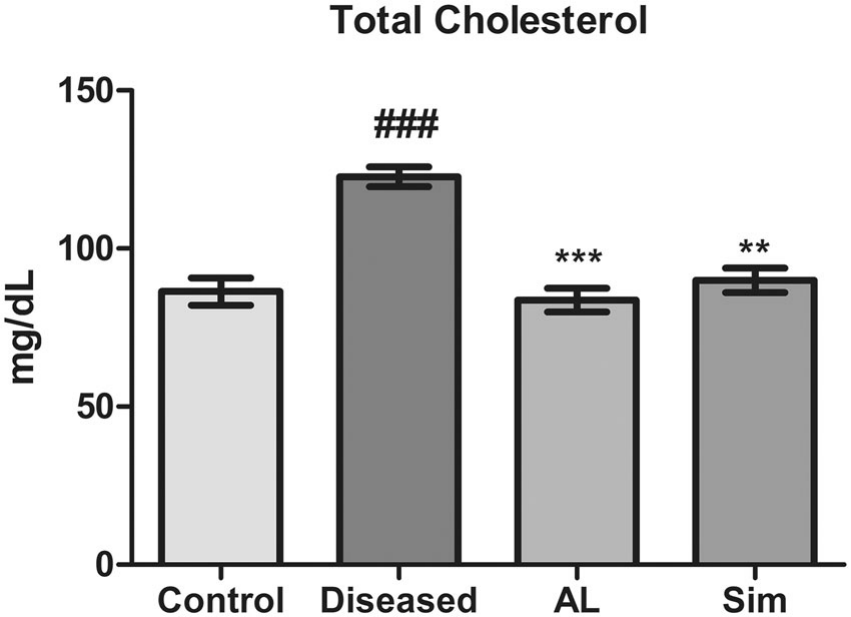
Graphical representation of mean ± SD relative total cholesterol levels in all groups (*n* = 6). *** = *p* ≤ 0.001 and ** = *p* ≤ 0.01 indicate significant difference in comparison to the diseased group while ### = *p* ≤ 0.001 indicates significant difference in comparison to the control group.

A significant increase in total cholesterol levels of the diseased group was observed as compared to the control (122.7 ± 5.450 vs. 86.38 ± 7.554) which means high-fat diet in the diseased group resulted in hyperlipidaemia. Methanol extract of *A*. *laookcha* leaves treated group showed a significant decrease in total cholesterol levels as compared to the diseased group (83.70 ± 6.414 vs. 122.7 ± 5.450). Similarly, the group treated with simvastatin also showed a significant reduction in total cholesterol levels when compared to the diseased group (89.93 ± 6.704 vs. 122.7 ± 5.450). When the individual effect of simvastatin and leaves extract of *A. lakoocha* was compared with one another, we found that both have almost similar potential to reduce total cholesterol levels (Figure 1).

### Effect of *Artocarpus lakoocha* leaves extract on HDL levels

**Figure 2.**
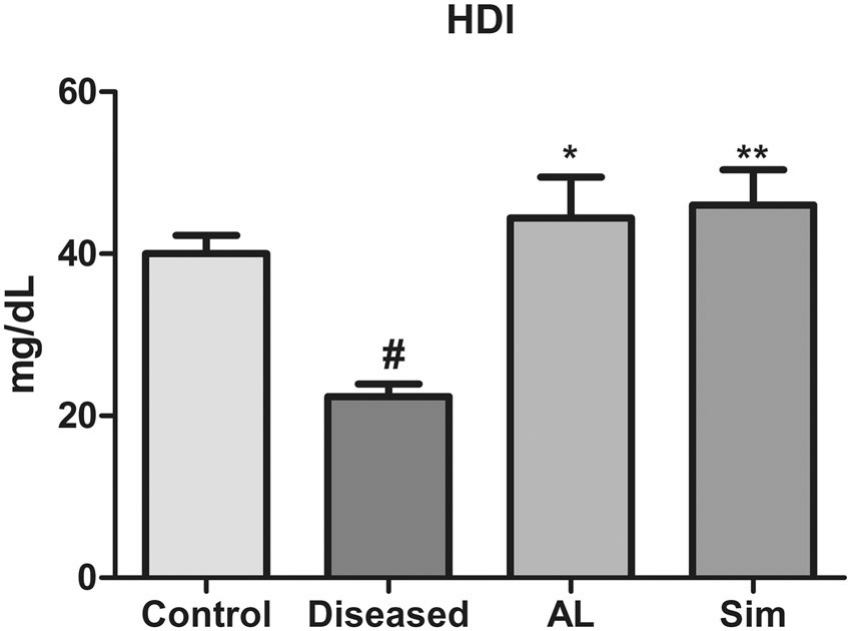
Graphical representation of mean ± SD relative HDL levels in all groups (*n* = 6). ** = *p* ≤ 0.01 and * *p* ≤ 0.05 indicate significant difference in comparison to the diseased group while # = *p* ≤ 0.05 indicates significant difference in comparison to the control group.

A significant fall in HDL levels of the diseased group in contrast to the control (22.35 ± 2.724 vs. 40.05 ± 3.875) was observed. Treatment with methanol leaves extract of *A. lakoocha* and simvastatin showed a significant rise in HDL levels when compared with the diseased group (44.42 ± 8.797 vs. 22.35 ± 2.724 and 46.05 ± 7.489 vs. 22.35 ± 2.724, respectively) (Figure 2).

### *Artocarpus lakoocha* leaves extract on LDL levels

**Figure 3.**
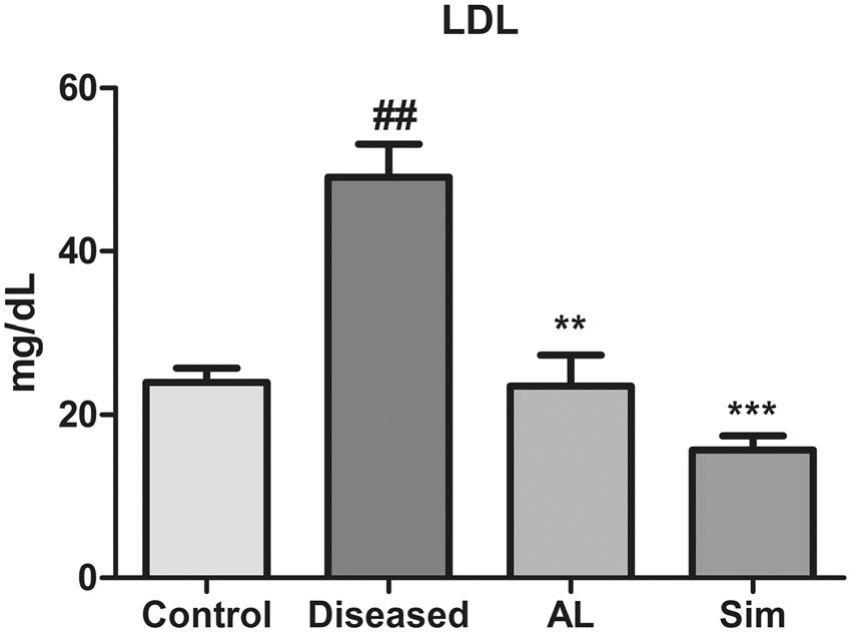
Graphical representation of mean ± SD relative LDL levels in all groups (*n* = 6). *** = *p* ≤ 0.001 and ** = *p* ≤ 0.01 indicate significant difference when compared to the diseased group while ##= *p* ≤ 0.01 indicates significant difference in comparison to the control group.

The results depicted a significant increase in LDL levels of the diseased group when compared to the control (49.06 ± 7.066 vs. 23.97 ± 3.063) which means high-fat diet resulted in raised LDL levels and caused hyperlipidaemia. Treatment with methanol extract of *A*. *lakoocha* leaves and simvastatin showed a highly significant decrease in LDL levels as compared to the diseased group (23.55 ± 6.545 vs. 49.06 ± 7.066 and 15.66 ± 3.046 vs. 49.06 ± 7.066, respectively). When the individual effect of methanol extract of *A*. *lakoocha* leaves and simvastatin were compared with one another in decreasing LDL levels, there was no significant difference found between them (Figure 3).

### Effect of *Artocarpus lakoocha* leaves extract on triglycerides levels

**Figure 4.**
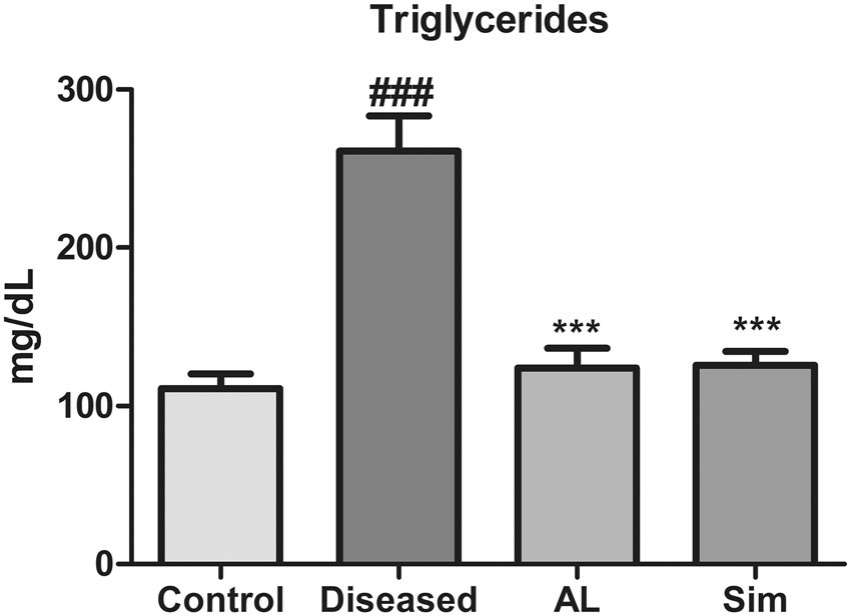
Graphical representation of mean ± SD of triglycerides levels in all groups (*n* = 6). *** = *p* ≤ 0.001 indicates significant difference when compared to the diseased group while ### = *p* ≤ 0.001 indicates significant difference in comparison to the control group.

A significant rise in triglycerides of the diseased group was observed in comparison to the control group (261 ± 38.735 vs. 111 ± 16.26). Treatment with methanol leaves extract of *A*. *lakoocha* and simvastatin exhibited a significant fall in triglycerides as compared to the diseased group (124.167 ± 21.7937 vs. 261 ± 38.735 and 125.83 ± 15.144 vs. 261 ± 38.735, respectively). While there was no significant difference observed in triglyceride reducing capacity of *A. lakoocha* leaves extract and simvastatin when compared with one another (Figure 4).

### Effect of *Artocarpus lakoocha* leaves extract on mRNA expression levels of squalene synthase

**Figure 5.**
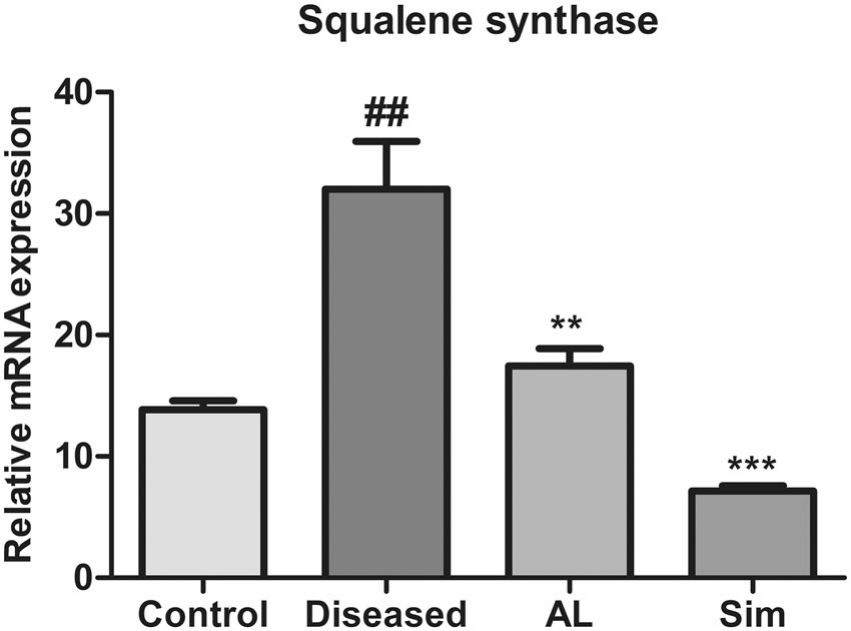
Graphical representation of mean ± SD relative mRNA expression levels of squalene synthase in all groups (*n* = 6). *** = *p* ≤ 0.001 and ** = *p* ≤ 0.01 indicate significant difference when compared to the diseased group while ### = *p* ≤ 0.001 indicates significant difference when compared to the control group.

The results showed that there was an upregulation of mRNA expression level of squalene synthase in the diseased group when compared to the control group (31.91 ± 5.292 vs. 13.34 ± 1.003). Treatment with methanol extract of *A*. *lakoocha* and simvastatin considerably decreased mRNA expression level of squalene synthase as compared to the diseased group (17.45 ± 2.48 vs. 31.91 ± 5.292 and 7.148 ± 0.76 vs. 31.91 ± 5.292, respectively) (Figure 5).

### Effect of *Artocarpus lakoocha* leaves extract on mRNA expression levels of HMG-CoA reductase

**Figure 6.**
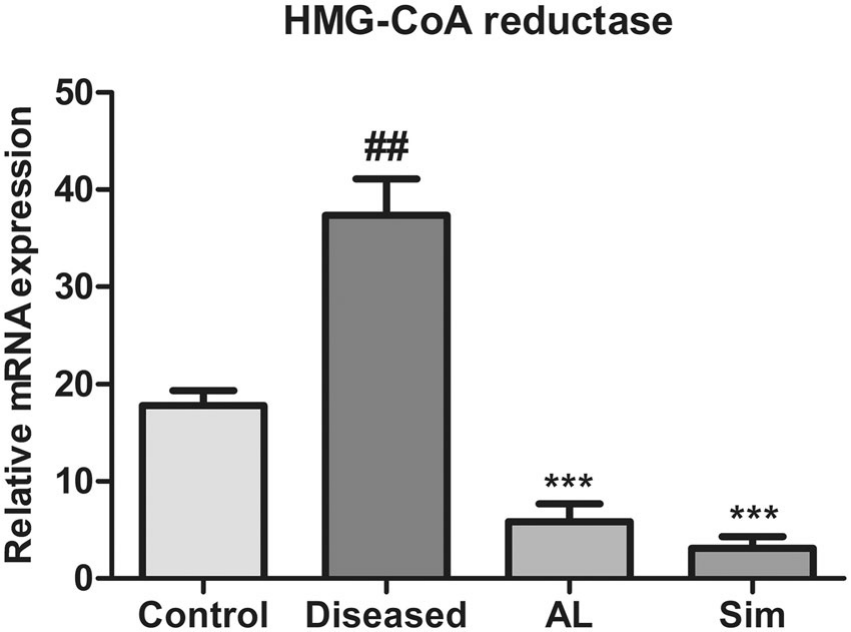
Graphical representation of mean ± SD relative mRNA expression levels of HMG-CoA reductase in all groups (*n* = 6). *** = *p* ≤ 0.001 indicates significant difference when compared to the diseased group while ## = *p* ≤ 0.01 indicates significant difference when compared to the control group.

The results showed that there was a significant upregulation of mRNA expression level of HMG-CoA reductase in the diseased group when compared to the control group (37.37 ± 6.492 vs. 17.796 ± 2.702). Treatment with methanol leaves extract of *A. lakoocha* and simvastatin considerably downregulated mRNA expression level of HMG-CoA reductase as compared to the diseased group (5.85 ± 3.164 vs. 37.37 ± 6.492 and 3.098 ± 2.09 vs. 37.37 ± 6.492). There was no significant difference found between *A. lakoocha* leaves extract and simvastatin in reducing mRNA expression of HMG-CoA reductase when compared with one another (Figure 6).

## Discussion

Hyperlipidaemia induced animals are worthy *in vivo* models for carrying out investigations on cholesterol homeostasis and drug trials and also helpful for examining the correlation of cholesterol metabolism disorders, atherogenesis, and consequently for the likely management of higher circulatory cholesterol levels (Otunola et al. 2010).

There is a close association between atherosclerosis and elevated or diminished serum lipids, particularly elevated LDL levels considered as a risk factor and decreased HDL level a protective factor (Zhu et al. 2008). Increased generation of oxidised LDL primarily contributes to the vascular damage related to elevated cholesterol levels (Saghir et al. 2012; Wen et al. 2019). Treatment with methanol extract of *A. lakoocha* leaves and simvastatin showed that there was a significant reduction of serum LDL levels. These findings are in parallel with various previous studies in which they demonstrated that dietary *Platycodon grandiflorus* (Jacq.) A. DC. (Campanulaceae) root powder and leaves extract of *Eclipta prostrate* Linn (Asteraceae) markedly reduced the LDL levels as compared to those of the hyperlipidemic control groups (Kim et al. 1995).

Epidemiological and clinical studies have also acknowledged the association of decreased HDL cholesterol concentration and an elevated likelihood of CVD (Assmann and Gotto 2004; Yokozawa et al. 2006; Adaramoye et al. 2008). The cardioprotective role of HDL has been attributed to its role in reverse cholesterol transport, its effects on endothelial cells, and its antioxidant activity (Nofer et al. 2002; Assmann and Gotto 2004). Our findings are correlated with previous studies in which it was found that hyperlipidemic animals when treated with methanol extract of *Vernonia amygdalina* Del. (Asteraceae) leaves showed significantly elevated plasma HDL cholesterols when compared with the diseased group (Adaramoye et al. 2008).

Several studies have reported a long-standing association of triglyceride and cholesterol levels in the prediction of CHD (Sarwar et al. 2007; Harchaoui et al. 2009). In an elegant *post hoc* analysis of the Helsinki Heart Study, it was established that cholesterol levels couldn’t be the sound predictor of CHD when elevated triglyceride levels are not present. Similar relations were also reported for individuals in the Prospective Cardiovascular Munster (PROCAM) study. These descriptions have been broadly quoted as proof that triglyceride measurement plays a vital role in the clinical assessment of CHD risk (Avins and Neuhaus 2000). These findings are similar to the studies done before in which it was found that oral administration of total flavonoids of *Perilla frutescens* Britton (Labiatae) leaves to hyperlipidemic rats was highly useful in decreasing the levels of serum total cholesterol and triacylglycerols. Another study also indicates that the ethanol extract of *Iris germanica* Linnaeus (Iridaceae) significantly reduced the lipid components which include cholesterol and triglycerides (Choudhary et al. 2005).

Squalene synthase enzyme catalyses the first committed step in the *de novo* cholesterol biosynthesis. Squalene synthase inhibitors reduce LDL cholesterol in circulation by upregulating the expression of hepatic LDL receptors in a way similar to statins (Tavridou et al. 2008). HMG Co-A reductase, a transmembrane protein, is of primary importance in the biosynthesis of lipids and is documented as the enzyme involved in the rate-limiting step in cholesterol biosynthesis, indicating the chief target for the cholesterol-lowering agents, i.e., statins (Istvan and Deisenhofer 2001; Kim et al. 2016). Simvastatin is the competitive inhibitor of HMG-CoA reductase (Xu et al., 2014). Our results showed the downregulation of mRNA expression levels of HMG-CoA reductase when treated with methanol leaves extract of *A. lakoocha.* These findings are in parallel to a previous study in which mRNA expression of HMG-CoA reductase was decreased when treated with a combination of four herbs (HVC1) and simvastatin (Kim et al. 2016).

## Conclusions

The outcome of our study shows that *A. lakoocha* leaves methanol extract at the dose of 250 mg/kg body weight, significantly decreased the total cholesterol, LDL, and triglycerides levels while significantly raising HDL levels in the hyperlipidemic rat model along with downregulation of mRNA expression levels of HMG-CoA reductase and squalene synthase. Further studies should be conducted to find out the most effective active ingredient of *A. lakoocha* leaves extract and the exact mechanism due to which it exerts its effect on cholesterol synthesis pathway.

## Data Availability

The data used to support the findings of this study are available from the corresponding author upon request.
